# Modelled optimisation approaches for laser cutting sheets simultaneously applied to EV component production

**DOI:** 10.1371/journal.pone.0275966

**Published:** 2022-10-21

**Authors:** Nathan Dodd, Russell Goodall, Erica Ballantyne, Graeme Heron

**Affiliations:** 1 Department of Mechanical Engineering, The University of Sheffield, Sir Frederick Mappin Building, Sheffield, United Kingdom; 2 Sheffield University Management School, The University of Sheffield (UK), Sheffield, United Kingdom; 3 Industrial Doctoral Centre in Machining Science, Advanced Manufacturing Research Centre with Boeing, University of Sheffield, Rotherham, United Kingdom; 4 Dept Materials Science & Engineering, The University of Sheffield, Sir Robert Hadfield Building, Sheffield, United Kingdom; Beijing Institute of Technology, CHINA

## Abstract

This paper proposes that laser cutting has potential as a viable alternative to stamping for mass manufacture of thin steel components such as stator and rotor components in the electric automotive sector. Current laser cutting processes are much less efficient than stamping. However, laser cutting is much more flexible and is used for small batches and one-off production. This paper assesses the potential of performing laser cutting operations of multiple sheets or layers simultaneously. This method is referred to herein as polystromata cutting. A numerical model is used to assess the manufacturing performance of stamping, traditional laser cutting and polystromata laser cutting. Polystromata laser cutting is shown to be capable of producing parts at 37% less cost than stamping. However, polystromata remains slower than stamping, taking 79% more time to produce each stator stack. Through this research it has been identified that optimisation of polystromata processes is more complex and performance efficiency varies wildly dependent on manufacturing set-up. This work aims to provide a best practice optimisation methodology for polystromata laser cutting by assessing results using different manufacturing objectives.

## 1 Introduction

The electric vehicle (EV) market is performing exceptionally well despite global economic conditions with sales rising by 43% despite overall car sales falling by 20% in 2020 [1]. Forecasters predict that EV sales could increase by as much as fourfold from 2018 sales Figs to 2023 [2]. Evidence is emerging from companies in the global electric motors market of moves toward higher levels of automation and increased uptake of novel technology [3, 4]. The cost of developing EV technologies is significant [5] and whilst the EV market continues to mature, companies will have to make difficult choices with regards to making these substantial investments or allowing competitors to gain first mover advantage [6].

An electric motor is made from four key components: stator, rotor, winding and shaft. The stator and rotor are generally made from electrical steels and are typically stamped. Advanced manufacturing techniques are being developed for electric machine components, such as the use of photochemical machining [7] and the production of stators by additive manufacturing [8]. Sheet material is typically delivered as a roll, referred to as mother coil (MC). The sheet is loaded onto a roller where it is then flattened and fed into a stamping machine. The cut sheet sections are then annealed. The stator is then assembled by collecting the punched sheets then, stacking, compression and joining, which seals the stack together. The stator slots are then electrically insulated by paper-like sleeves and the copper windings are then installed into the stator [9, 10].

The rotor component is produced by stacking punched sheets in the same way as assembling the stator, this is known as a transversally laminated rotor. Other rotor designs exist, such as the axially laminated rotor which require alternative manufacturing and assembly processes to produce the rotor as it is not produced from standard laminations. Further assembly processes are undertaken, including the installation of a shaft into the rotor and the housing unit being assembled around the stator. Research by Niazi [11] demonstrates how different rotor designs can be used to reduce cost and increase ease of manufacture for synchronous reluctance applications.

The use of laser cutting as a prominent method of manufacture for components such as *stators* and *rotors* is an area of production strategy which has remained undeveloped due to the established use of stamping and the perceived technological limitations of laser cutting from a manufacturing efficiencies perspective. Stamping requires a high level of initial investment owing to the manufacture of dies, but once set up, is a relatively quick and consistent method. Laser cutting is a much more time-consuming process but offers vastly superior flexibility and as such is a preferred method for producing small batches or one offs [12]. Whilst other cutting processes exist such as abrasive water jet and wire cut electric discharge machining, these are typically used for specific applications, such as cutting very thin sheets where time is not an important constraint [13].

This paper proposes and develops a novel manufacturing process for performing simultaneous cutting operations with sheet metal. Layers of sheet material can be stacked and cut in a single laser cutting operation. The act of cutting multiple laminates in a single operation is henceforth referred to as *polystromata*. The productivity of laser cutting could be increased significantly by performing a polystromata cutting process and research is beginning to be published which considers the effects of cutting sheets simultaneously in a single operation, noting further applications in automotive industry if the process can be sufficiently optimized [14].

The aim of this research is to develop an optimisation strategy for this novel polystromata laser cutting technique, which is designed to be compatible with complex automotive platforms and in keeping with wider operations strategy objectives. A complex manufacturing model has been used in this paper to represent the conditions of producing stator components for an EV machine (power unit). The model developed in this research uses data sciences techniques which are already established in the field of manufacturing optimisation and laser cutting applications [15–17]. The model in this research is intended to identify the compromises which occur in the optimisation process and to develop an optimal manufacturing strategy. Furthermore, an assessment is made to identify areas of opportunity within that strategy using advanced automation and technology techniques.

## 2 Modelling methodology

A complex production model has been developed to consider the operational effectiveness of cutting stacks of material for EV machine production. The EV components considered are the rotor and stator components, which are manufactured by stacking cut sheets of electrical steel. The laser cutting model consists of various subsections, containing elements relating to; production quantities, part design, manufacturing set-up, manufacturing process and activity-based cost allocation. The activity-based costing approach allocates resources, such as costs, time, and labour to an activity rather than a specific product [18]. This focus on processes rather than product ideal as in this case, the size, style and materials used in the final product are flexible. Whilst it is possible to track direct labour and material costs in an activity-based costing model, the use of an overhead allocation system to define and absorb the range of costs which occur in the manufacturing system separates activity-based costing from traditional costing methods [19, 20]. Additionally, a Multi-objective optimisation problem solving approach [21] is applied in this research to a laser cutting manufacturing platform for EV machine components. Multi-objective optimisation has been used previously in manufacturing to identify process parameters and set-ups [22, 23] and is used similarly, with a greater view towards economic outputs.

The time taken to produce the total quantity of stacks, *t*_*α*_, is given by Eq 1, where *n*_*lams*_ is the total required laminates, *α*_*O*_ is the cutting operations per minute, *β* is the laminates cut per operation, *t*_*L*_ is the part change over time, *n*_*O*_ is the number of operations, *t*_*J*_ is the time taken to perform maintenance on the laser cutting machine, *n*_*J*_ is the number of operations per maintenance requirements, and *n*_*L*_ is the number of laser cutting machines used during production. The number of operations per minute (Eq 2) is a function of the cutting speed, *U*_*L*_, and length of cut, *L*_*C*_,. The laser cutting speed (Eq 3) is derived from a known laser cutting capability [24] relative to depth of material being cut. The cutting stack depth is found using an optimisation process (explained in detail in section 2.1). The time taken to produce a stack through stamping is proportional to the stamping rate, which is modelled at 300 strokes per minute. The model includes activities such as maintenance downtime for stamping, resulting in a stack production rate at volume of around 112 seconds per stack.


$$t_{\alpha }=\frac{\frac{n_{lams}}{\alpha_{O}.\;\beta }+t_{L}.n_{O}+t_{J}.\frac{n_{O}}{n_{J}}}{60.\;n_{L}}$$ (1)

$$\alpha_{O}=60.\frac{U_{L}}{L_{C}}$$ (2)

$$U_{L}=\frac{100-(4.241.\;L_{L}.\;1000)}{1000}$$ (3)

The total production costs are calculated by addition from various subsections. These include; Energy costs (Eq 4) which are found by considering the cost rate of energy, *C*_*eW*_, the energy usage rate, *E*_*P*_, and the time that energy is used for, *t*_*α*_. Machining costs (Eq 5) which are predicated on the cost of purchasing laser cutting machines, where *C*_*L*_ is the cost of purchase one machine. Maintenance costs (Eq 6) are predicted based on numbers of operations conducted, where *C*_*J*_ is the cost of performing a maintenance operation. Overhead costs (Eq 7) have been included to represent the cost of time taken to manufacture goods. The overhead rate, *C*_*t*_, is a simple method of capturing the cost of labour, space and other resources that would be used by the process, but not directly attributable. The overhead rate is highly dependent on the specific circumstance of any business but is a useful measure to understand the further effects of manufacturing time.


$$C_{E}=C_{eW}.E_{P}.t_{\alpha }$$ (4)

$$C_{\alpha o}=n_{L}.C_{L}$$ (5)

$$C_{\alpha m}=C_{J}.\frac{n_{o}}{n_{J}}$$ (6)

$$C_{o}=t_{\alpha }.C_{t}$$ (7)

**Fig 1:**
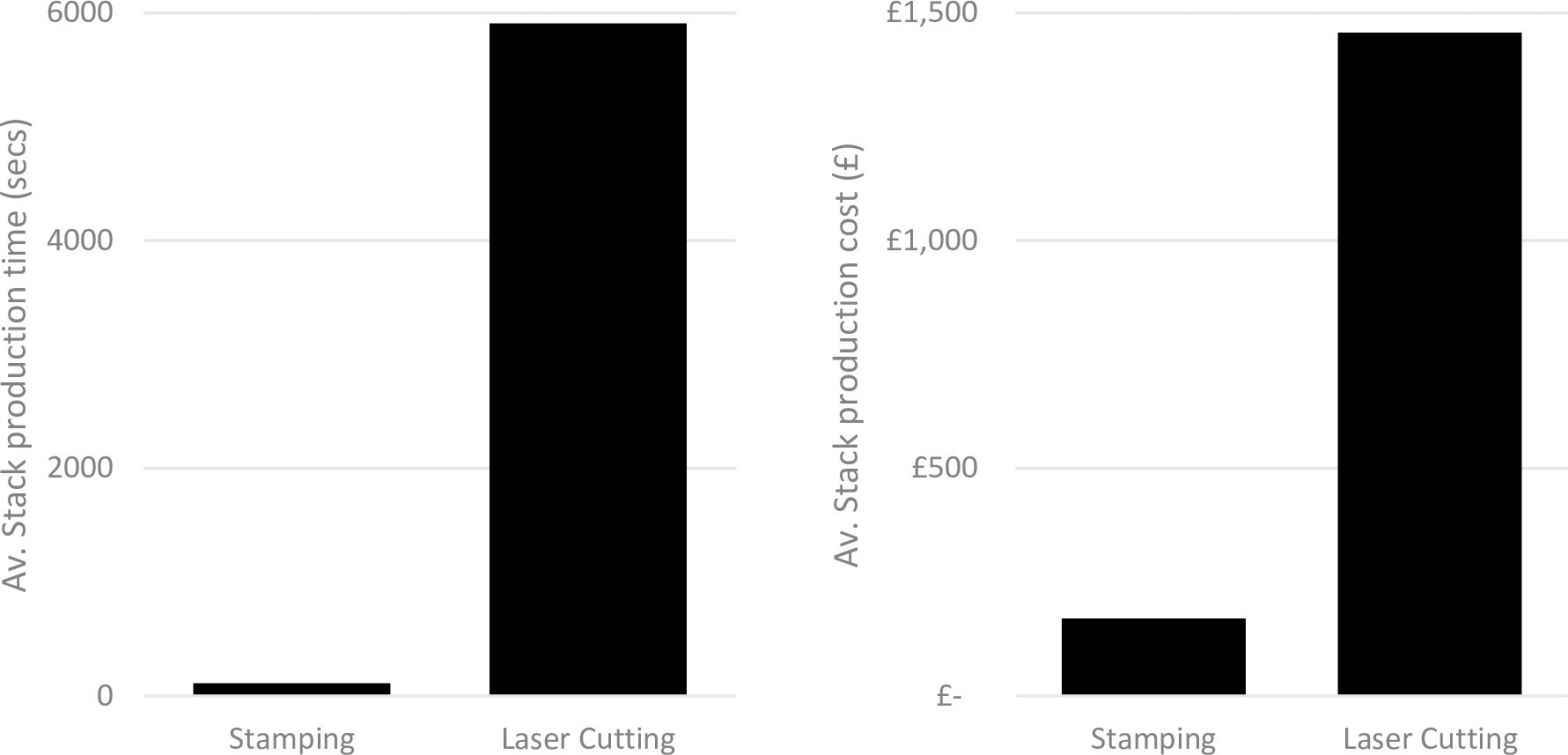
Comparison of Stamping and Laser cutting output based on current technology.

Initial findings from the model using the dataset in from the table in S1 Table show the current gap between stamping and laser cutting, where laser cutting takes considerably more time and cost to produce components compared to stamping (Fig 1). These results further outline why laser cutting is not currently considered for mass manufacturing operations. The polystromata method is expected to reduce this gap and make laser cutting a much more competitive alternative to stamping.

### 2.1 Optimisation process

**Fig 2:**
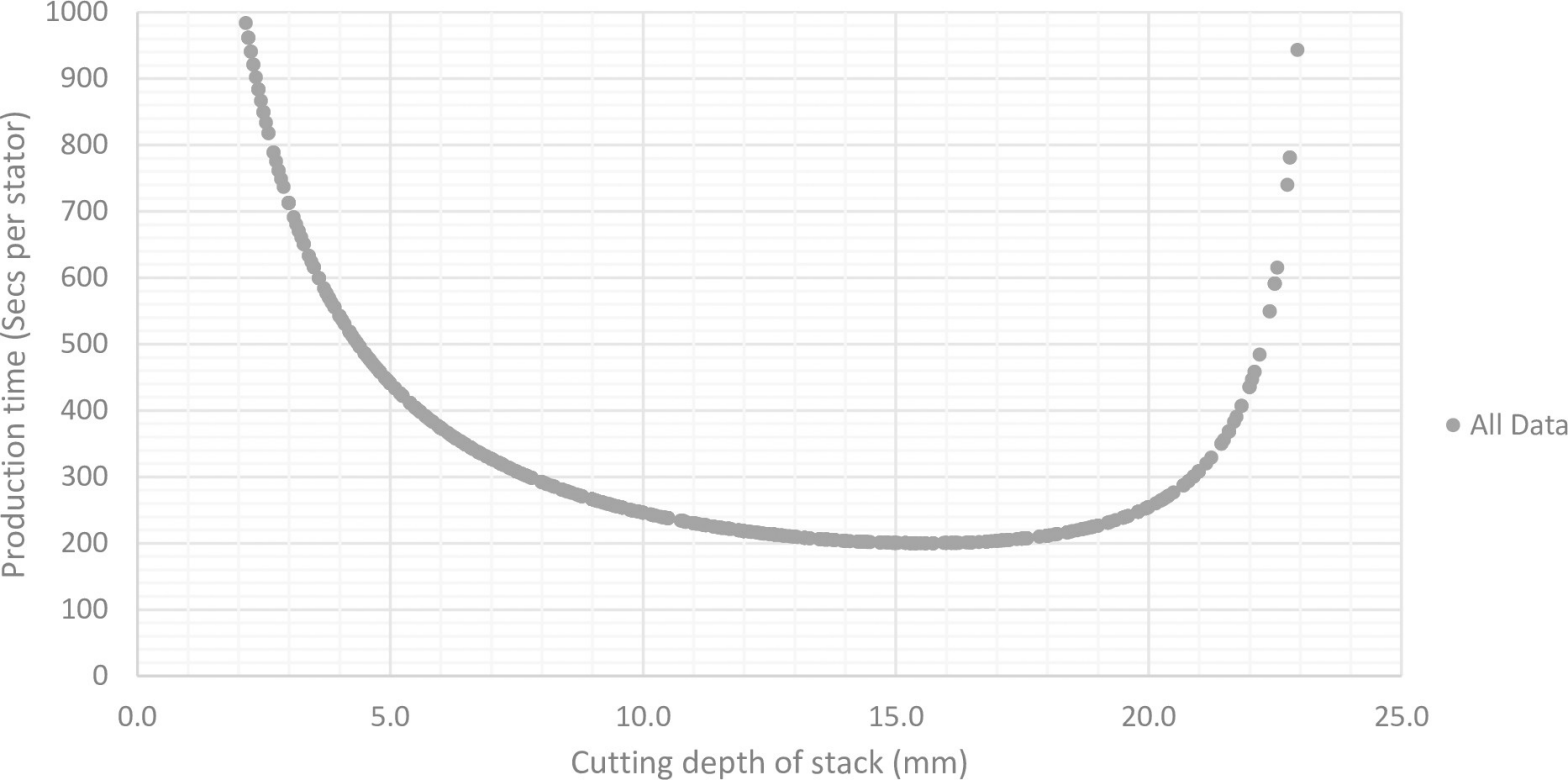
Stator production times for an unoptimized model.

One of the major challenges which occurs from cutting stacks of material is producing an efficient manufacturing set-up. Without using some form of optimisation procedure, the production times can vary greatly, as demonstrated by the range of possible results in Fig 2. The time taken to produce parts at volume through polystromata cutting is highly dependent on the time taken to cut a stack (with deeper stacks taking longer to be cut) and the time taken to complete activities between operations such as the physical movement of parts. This problem is true for any output of the system, such as production costs, material utilisation and so on.

The solution used in the model is to perform a 2-stage analysis of the dataset. The first stage of the process uses a broad range of combinations of laminate thickness (in the region 0.2mm to 0.5mm) and number of laminates cut in a single operation (ranging from one sheet to hundreds). The results are provided by a dynamic look-up table. This enables results to be updated automatically based on any set of parameters within the model. This first stage of the process calculates results with a limited precision, owing to the discrete input of parameters. The results of this first stage provide a target region where in stage 2 a very precise set of combinations for laminate thickness and number of laminates cut are analysed.

**Fig 3:**
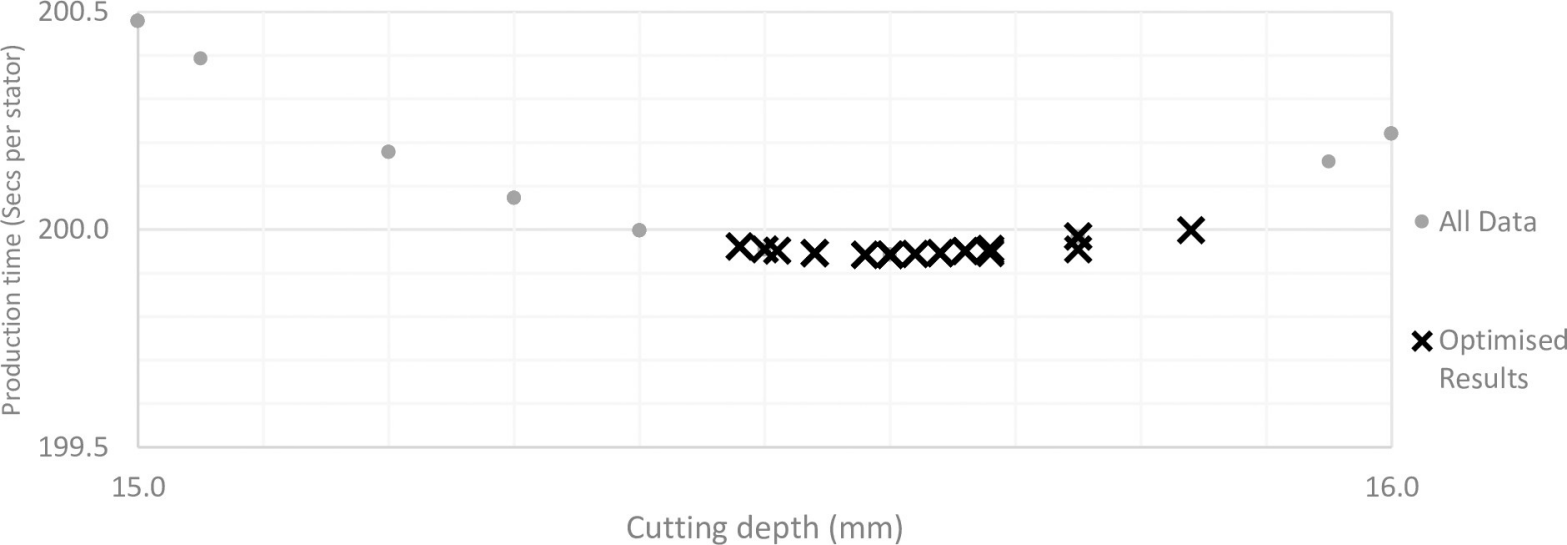
Stator production times for an unoptimized model and a time optimised model.

For parameter set 1, the best production times occur when there is a cutting depth somewhere in the region of 15mm-16mm. Using this knowledge, a more defined search is conducted, where for a given sheet thickness, results are only considered where the number of laminates cut per operation equate to cutting depths in the search region. Using this method, the model is capable of returning only the most efficient results, based on the optimisation criteria, as demonstrated by the optimised results in Fig 3.

The model can either be optimised to provide the most efficient production times for a given set of parameters, or alternatively, to provide the most cost-efficient production set-up. Using the same optimisation process as before, results were obtained across a range of sheet thicknesses to assess the differences in optimisation choice.

Production costs have been calculated using a variety of overhead rates to reflect the potential discrepancies in applying the data to different sized organisations. Overhead rates are directly proportional to time, and so it is useful to understand how they might affect the optimisation decision process as a result.

A further study was conducted using a different set of parameters, considering the effect of a much greater gas cost per operation and increased maintenance costs. These parameters are considered here because they are directly related to costs per operation, and both parameters are included in the model with assumed cost rates. It is not necessarily that gas costs and maintenance costs specifically are expected to be much higher, but that their implementation in the model allows them to effectively be used as a way of economically controlling ‘operations costs’.

## 3 Results

Parameter set 1 represents the original data set which the model has been developed with. Parameter set 2 uses the same data, except for those changes listed in Table 1.

**Table 1:** Parameter sets used in laser cutting study.

	Parameter	Units	Parameter set 1	Parameter set 2
**L**	Maintenance Cost	£	2000	5000
**L**	Gas cost	£	0.05	2.00

**Fig 4:**
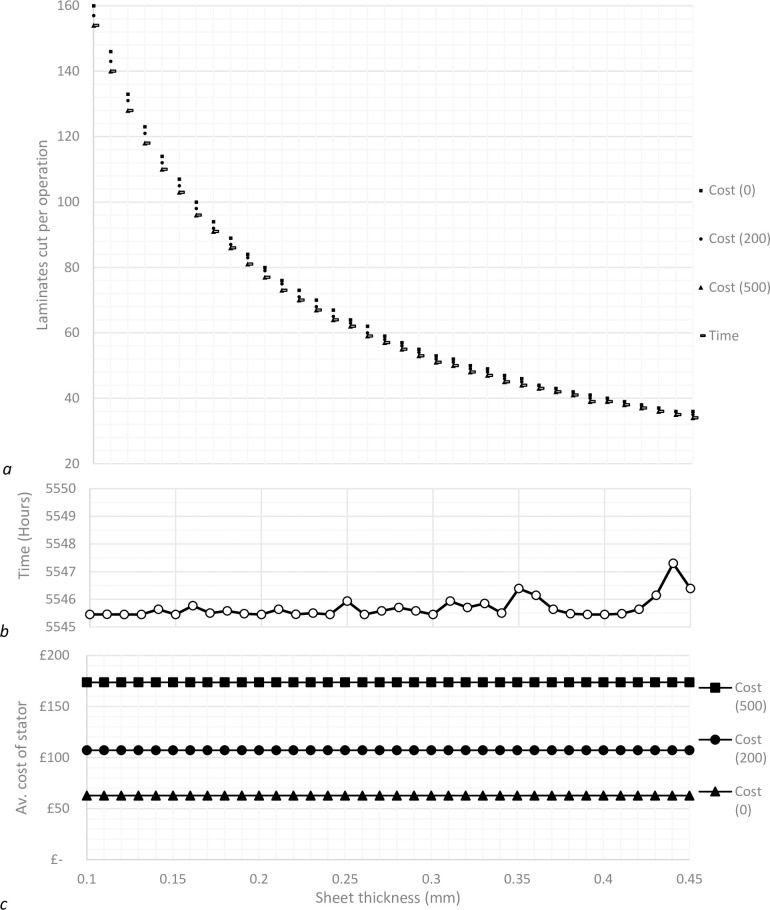
Comparison of optimisation processes, where gas price is £0.05 and maintenance costs £2000.

The results in Fig 4 show that the difference in production settings produce a small, but noticeable difference between each data set. Differences are more pronounced when sheet thickness is thinner, which is expected as more sheets are required to create the same changes in cutting depth. It can be seen that as overhead rates increase, the optimisation results become synchronised. This makes sense, as with increased overhead rates, there is increased cost associated with time, and as such, optimising costs tends towards optimising time as overhead rates increase.

It should be noted that because the model is using an optimisation procedure, the variations in the results relative to the range of manufacturing set-ups tested are very small, regardless of whether the model is optimised for minimum cost or time, as demonstrated by the optimised results in Fig 3. It may be the case however that with a different set of parameters, most likely those relating to a specific laser cutting machine, there may be much more variation in results across the range of possible cutting set-ups.

### 3.1 Parameter set 1

**Fig 5:**
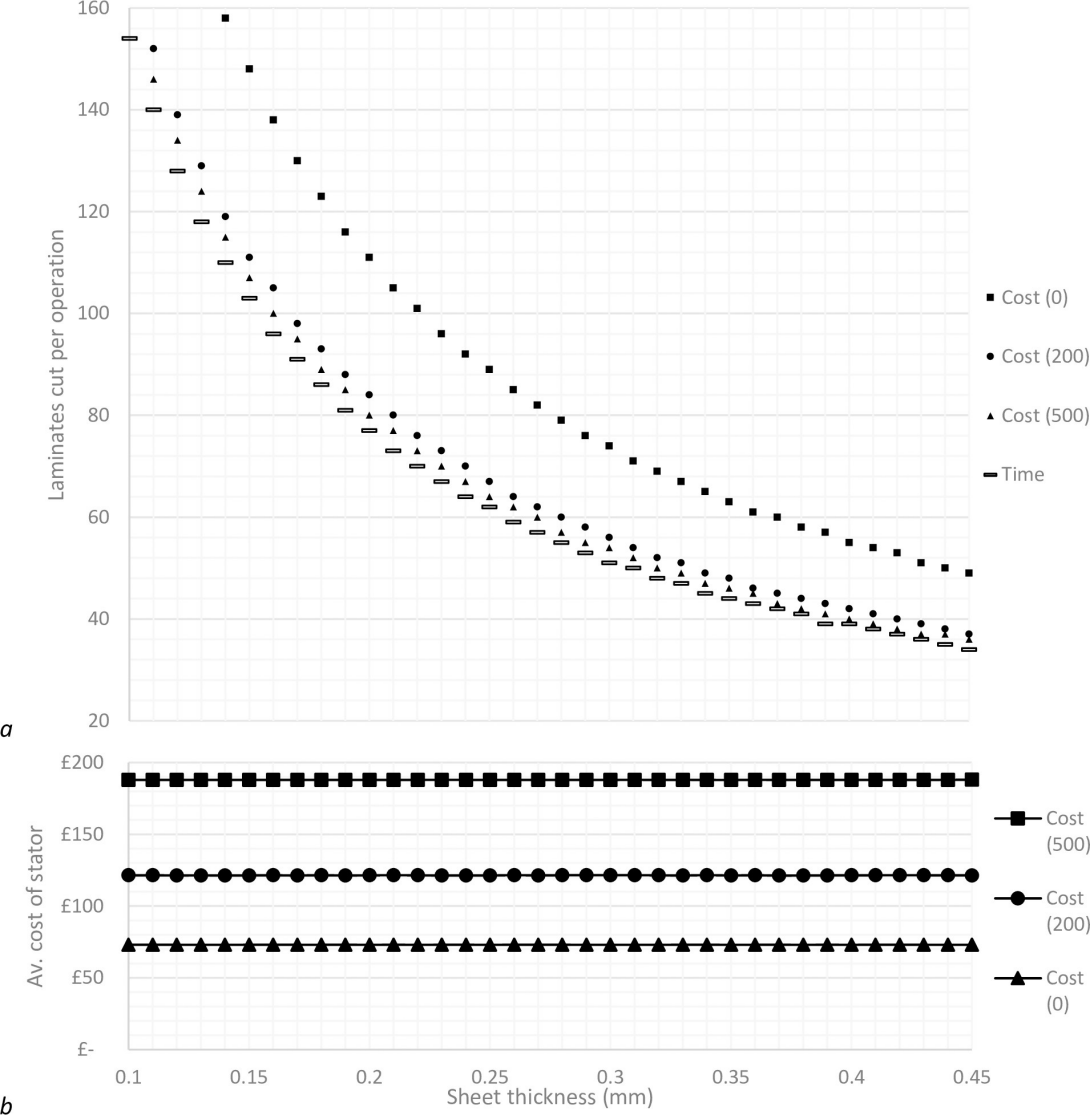
Comparison of optimisation processes, where gas price is £2.00 and maintenance costs £5000.

The alternative parameter set shows results with a much greater spread (Fig 5) than those initially gained from the starting set of assumptions (Fig 4). The time optimised results are exactly the same, which is expected as the parameters which have been changed related to cost functions only. The same trend occurs where increased overhead rates cause the cost optimised results to tend towards the time optimised results, however, in this case a much greater overhead rate is required to create parity with the time optimised results. It remains true that for any cutting set-up, there are only very small deviations between set ups regarding the optimised output.

### 3.2 Parameter set 2

The greatest differences can be seen when the overhead rate is set to £0. Whilst an overhead rate of £0 is unrealistic, it provides an extreme case and is a very useful baseline for comparison where actual overhead rates are unknown, such as during the R&D phases of production research. This method also aligns with other analytical approaches, such as quality function deployment [25]. The overhead rate can have a significant impact on the decision to invest in new machinery and equipment. For example, if investing in an automated loading machine, an increased overhead rate would create greater need to reduce production times and further incentivise the purchase. However, the new machine would also incur some alteration to overhead rates as its factory footprint, labour and skills requirements, and other factors would need to be taken into consideration. The skills required to operate the more advanced technology may be difficult to source or not readily available, which increases costs and risks in transitioning towards more advanced processes. It is also possible to use the introduction of a new manufacturing facility as an opportunity to optimise the current work space and layout design, such as creating a leaner work piece route or reducing work in progress inventory. Additional benefits may exist in terms of the effect on the work force, as a reduction in manual handling stress may result in improvements within the socio-technical domain.

**Fig 6:**
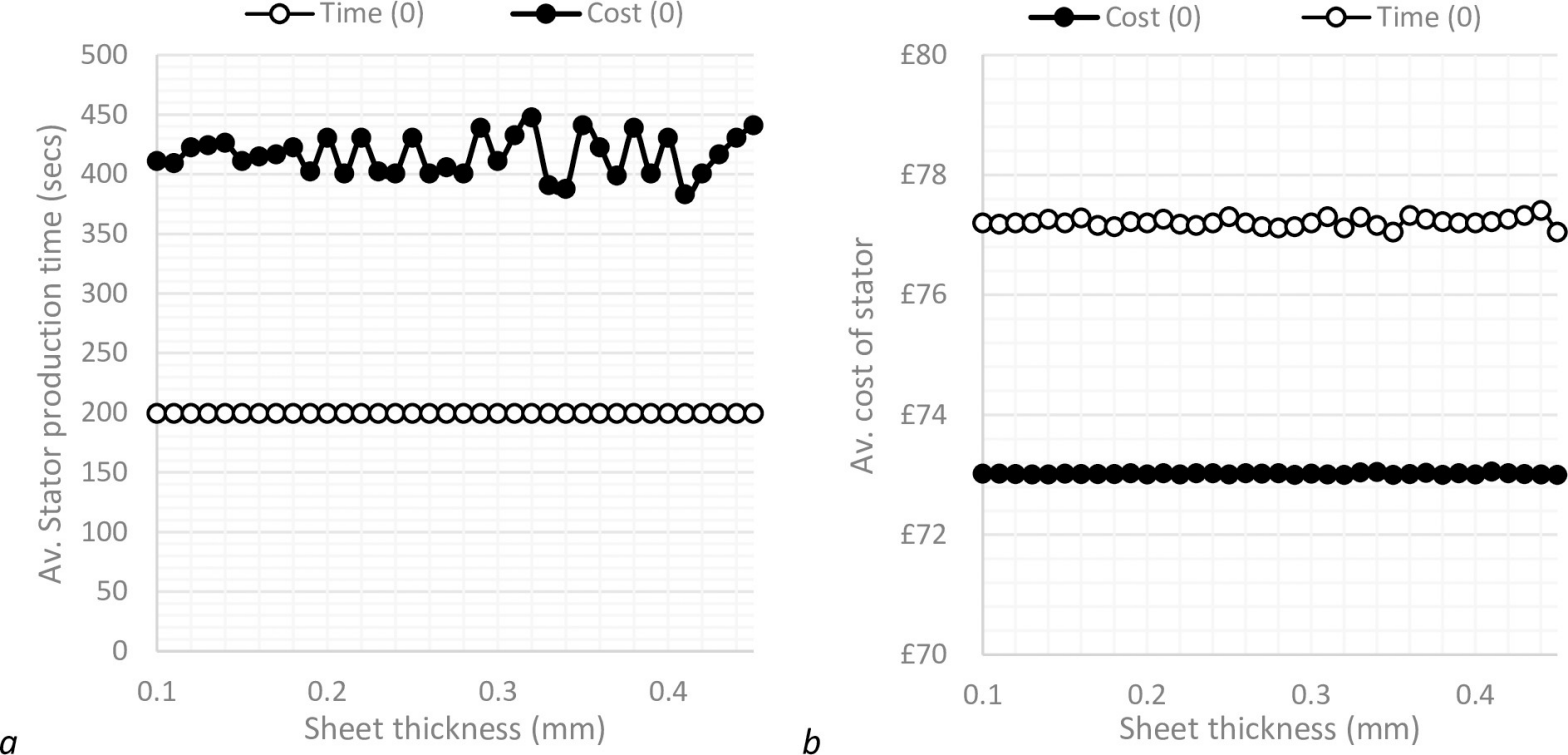
Comparison of manufacturing effectiveness for a time optimisation and cost optimisation approach where overhead rate is £0.

In this case, the two different optimisation processes provide very different outcomes. Optimising for minimal production time results in an increase of average stator costs by approximately 6% compared to the cost optimised set-up. Alternatively, optimising the model to reduce costs dramatically increases production times by approximately 100% in the best case. Interestingly, Fig 6A shows an increased variance in production time results across the range of cutting set-ups when the model is cost optimised.

This study shows the importance of having a statistical process control system in place for the implementation and use of a laser cutting production process, particularly if the process is expected to perform cuts of multiple sheets per cutting operation, as is examined here. As well as the cost and time aspects of this process, there is a question reflecting the quality of cut produced when a stack cut is performed. Research in this area is beginning to be conducted with results showing a slight reduction in electromagnetic performance for polystromata cut parts relative to stamping [26]. Whilst the research herein is limited to providing performance related data, the model and optimisation processes developed here are easily adaptable to consider these effects. In the future it is possible that this method of optimisation can be used to produce production set-ups which optimise time, cost, and quality trade-offs, bespoke to the requirements of the platform operator. Further research is required to develop a detailed understanding of how cut quality might vary relative to individual manufacturing parameters, such as cutting speeds, forces, and materials. An extensive production trial would be needed to produce such data.

**Fig 7:**
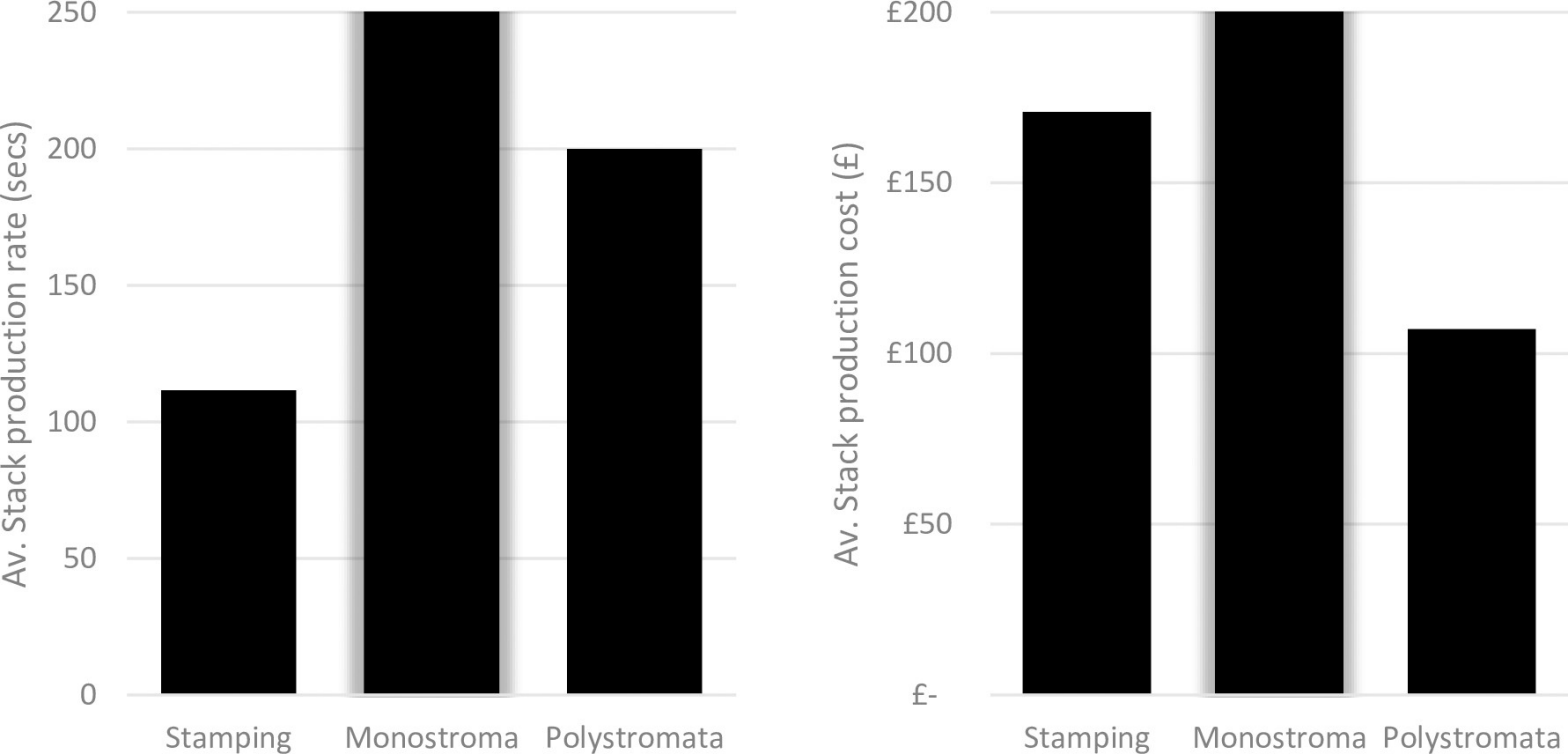
Comparison of production rates and costs for stamping and laser cutting of stator stacks.

Fig 7 clearly shows that the optimised polystromata laser cutting method presents a much more competitive alternative to stamping than basic monostroma laser cutting can achieve. Polystromata laser cutting offers a potential to reduce costs of 37%, where stamping involves the use of expensive tooling which are used during the cutting operation. stamping remains the most time efficient method, with polystromata taking 79% longer to provide each stack. The biggest costs in laser cutting are related to the investment required in purchasing the laser cutting machines. Nonetheless, volume increases may benefit depreciation models, forecasting and the ability to become more agile around customer demands, which in turn, increases the intangible asset value of the stators themselves from amortisation perspectives related to accounting periods.

## 4 Conclusions

The automotive industry is currently presented with an opportunity to develop new, lean manufacturing platforms in the electric vehicle market. One of these developments is the use of novel laser cutting techniques in the mass manufacture of machine laminations. One of the key findings of this research is the demonstration of polystromata laser cutting as a real alternative to stamping in the production of thin sheet components such as stator and rotor laminations. By applying a considered optimisation process to the modelled polystromata platform, it was possible to achieve a manufacturing output at lower costs per unit (stator stack) than stamping and to dramatically reduce production times compared to basic monostroma laser cutting methods (Fig 7). It is expected that with further research and development that the manufacturing output for polystromata processes would continue improve given its suitability for incorporation of lean and agile operations and the further scaling up of manufacturing volumes.

The results in this research indicate that in most cases the best polystromata laser cutting set-up is achieved using a time-oriented optimisation. Comparatively, cost optimisation is more time consuming to set up, and whilst production costs are slightly higher using a time optimisation objective, the overall manufacturing system performs more strongly as a result of the far superior production times. The overhead rate used in the activity-based costing approach also has an impact on the relative performance of time vs. cost optimisation. For instance, smaller businesses with lower overheads (which tend towards £0) will see greater benefits in cost optimising the laser cutting process, however as overheads increase (tending away from £0), the decision to time optimise increasingly becomes more beneficial.

Using the activity-based costing approach in combination with the optimisation processes in the model, it is possible to further assess the potential of upgrading production line technology. The results in this research demonstrate, for example, how increases in overhead rates as a result of investment into more expensive equipment can create substantial changes to the effectiveness of the production operation, particularly where the business has opted for a cost optimised approach.

The optimisation processes developed in this research are easily transferable to more general applications during design decision costing analyses in manufacturing systems. The assessments made in this paper have identified best case manufacturing set-ups for multiple objective criteria and can be expanded to consider any range of computable metrics.

## Supporting information

S1 Table Production model including stamping & laser cutting parameters. (XLSX)
